# Evolution of a highly functional circular DNA aptamer in serum

**DOI:** 10.1093/nar/gkaa800

**Published:** 2020-10-06

**Authors:** Yu Mao, Jimmy Gu, Dingran Chang, Lei Wang, Lili Yao, Qihui Ma, Zhaofeng Luo, Hao Qu, Yingfu Li, Lei Zheng

**Affiliations:** School of Food and Biological Engineering, Hefei University of Technology, Hefei 230009, China; Department of Biochemistry and Biomedical Sciences, McMaster University, Hamilton L8S4K1, Canada; Department of Biochemistry and Biomedical Sciences, McMaster University, Hamilton L8S4K1, Canada; School of Food and Biological Engineering, Hefei University of Technology, Hefei 230009, China; School of Food and Biological Engineering, Hefei University of Technology, Hefei 230009, China; School of Food and Biological Engineering, Hefei University of Technology, Hefei 230009, China; School of Life Sciences, University of Science and Technology of China, Hefei 230026, China; School of Food and Biological Engineering, Hefei University of Technology, Hefei 230009, China; Department of Biochemistry and Biomedical Sciences, McMaster University, Hamilton L8S4K1, Canada; School of Food and Biological Engineering, Hefei University of Technology, Hefei 230009, China

## Abstract

Circular DNA aptamers are powerful candidates for therapeutic applications given their dramatically enhanced biostability. Herein we report the first effort to evolve circular DNA aptamers that bind a human protein directly in serum, a complex biofluid. Targeting human thrombin, this strategy has led to the discovery of a circular aptamer, named CTBA4T-B1, that exhibits very high binding affinity (with a dissociation constant of 19 pM), excellent anticoagulation activity (with the half maximal inhibitory concentration of 90 pM) and high stability (with a half-life of 8 h) in human serum, highlighting the advantage of performing aptamer selection directly in the environment where the application is intended. CTBA4T-B1 is predicted to adopt a unique structural fold with a central two-tiered guanine quadruplex capped by two long stem–loops. This structural arrangement differs from all known thrombin binding linear DNA aptamers, demonstrating the added advantage of evolving aptamers from circular DNA libraries. The method described here permits the derivation of circular DNA aptamers directly in biological fluids and could potentially be adapted to generate other types of aptamers for therapeutic applications.

## INTRODUCTION

Aptamers are nucleic acids with well-folded tertiary structures to recognize a target molecule (1–3). Because of their excellent molecular recognition properties, biocompatibility and non-immunogenicity, aptamers hold the potential for diverse applications, including therapeutics and diagnostics (4–6).

A prerequisite for successful biomedical application of aptamers is their nuclease resistance and conformational stability in biological media. Standard nucleic acids are vulnerable to nucleases in biological media, which seriously hampers the practical applications of the aptamers derived from them (7). Chemical modifications are typically used to make aptamers nuclease-resistant after they are created by in vitro selection or SELEX (1–3). However, chemical modifications often require complicated organic synthesis, can reduce the activity of aptamers and produce undesired biological side effects (8–10). Circularization of a linear aptamer is an attractive alternative for several reasons. First, a circular construct becomes completely resistant to exonucleases, a primary source for DNA degradation in biological fluids, especially in blood. Second, circularization of a linear aptamer can increase its thermal stability. Moreover, circularization permits the use of natural nucleotides, which could avoid potential toxicity associated with chemical modification (11–14). Recently, Tan's group reported circular bivalent aptamers (cb-apt) by ligating two aptamer-based oligonucleotide sequences. Compared with the precursor aptamer, cb-apt has improved stability and better binding activity (15). Jia's group designed two double-strand circular aptamers for targeting circulating tumor cells (CTCs), resulting in enhanced ability to capture CTCs *in vivo* (16). Although a linear aptamer can be redesigned into a circular aptamer, additional nucleotides will have to be carefully selected and incorporated into new constructs to minimize the impact of circularization on the activity of the aptamer (17,18).

A large number of excellent linear DNA aptamers have been selected to bind important protein biomarkers. We believe that excellent circular DNA aptamers can be re-engineered from these linear aptamers to make them more desirable for applications in intended biological fluids. However, rational engineering of a circular DNA aptamer that is highly active in biological fluids from a known DNA aptamer represents a daunting challenge. Instead, we conceived an ‘adaptation’ strategy to achieve two tasks in one: converting a known linear aptamer into a circular aptamer and making it highly functional in an intended biological fluid. Since this is the first study of this nature, we chose to focus on TBA_15_, a widely studied 15-nucleotide (nt) DNA aptamer that is known to bind human thrombin with high affinity and specificity (19–22). Our objectives were to convert this linear DNA aptamer into a circular form with similar or improved properties and to make it highly functional in human serum.

Thrombin is a serine protease that plays a key role in blood coagulation, and its pivotal roles in coronary heart disease and other thrombotic disorders have precipitated the efforts toward identifying thrombin binders as anticoagulant agents (23–25). TBA_15_, with the sequence 5′-GGTTG GTGTG GTTGG-3′, was derived by SELEX for binding to human α-thrombin and has been shown to have attractive anticoagulation activity (26). A number of studies have been devoted to further improve the activity of this aptamer. For example, Mayer's group used a poly-dA linker to connect two aptamers which target distinct protein subdomains and the fusion aptamer displays 30-fold enhanced anticoagulant activity when compared to TBA_15_ (27). Soh's group reported a selection strategy to derive the most fitting linker sequence for the same aptamer pair and the derived bivalent aptamer shows 200-fold improvement in binding affinity (28). However, linear aptamers like these are susceptible to nuclease digestion, which seriously hinders their therapeutic applications. Placing TBA_15_ into a circular form and deriving a highly functional circular DNA aptamer were expected to overcome this issue.

## MATERIALS AND METHODS

### Oligonucleotides and other materials

All DNA oligonucleotides (Supplementary Table S1) were synthesized and purified with HPLC by Sangon Biotech Company, Ltd (Shanghai, China). T4 polynucleotide kinase (PNK), T4 DNA ligase, phi29 DNA polymerase, EcoRV, adenosine 5′-triphosphates (ATP) and deoxyribonucleoside 5′-triphosphates (dNTPs) were purchased from New England Biolabs (NEB, Beijing, China). γ-[^32^P]-ATP was acquired from Perkin Elmer (Woodbridge, ON, Canada). Human α-thrombin was purchased from Haematologic Technologies. The process of protein immobilization on magnetic beads (M-270, carboxylic acid functionalized, Life Technologies) was performed according to the manufacturer's protocol for two-step coupling using ethyl (dimethylaminopropyl) carbodiimide and N-hydroxysuccinimide (Sigma). LightCycler^®^ 480 high resolution melting master was purchased from Roche. Human serum was purchased from XinFan Biotech (Shanghai, China). Water was purified with a Milli-Q Synthesis from a Millipore system. All other chemicals were purchased from Sigma-Aldrich and used without further purification.

### Instruments

Absorbance measurements were recorded with a microplate reader (Tecan infinite M1000 Pro, Männedorf, Switzerland). The autoradiogram and fluorescent images of gels were obtained using Typhoon 9200 variable mode imager (GE Healthcare) and Bio-Rad image system. The intensity of each band was analyzed using Image Quant software (Molecular Dynamics). Thermal melting temperatures were measured by Roche LightCycler96 quantitative fluorescence PCR instrument. PT times were measured by using a semi-automatic coagulometer (Ruimai, Nanjing, China).

### Library preparation

The DNA library, denoted Lib, contains a 15-nucleotide thrombin binding aptamer flanked with two 20-nucleotide random domains and two constant domains at the 5′-end and 3′-end (note that the constant domain contains a recognition site for the restriction enzyme *Eco*RV). Circular DNA library was prepared from 5′-phosphorylated linear DNA oligonucleotides through template-assisted ligation with T4 DNA ligase. Each linear DNA oligonucleotide was phosphorylated as follows: a reaction mixture (50 μl) was made to contain 1 nM linear oligonucleotide, 20 U PNK (U: unit), 1× PNK buffer A (50 mM Tris–HCl, pH 7.6 at 25°C, 10 mM MgCl_2_, 5 mM DTT, 0.1 mM spermidine) and 2 mM ATP. The mixture was incubated at 37°C for 30 min, followed by heating at 90°C for 5 min. The circularization reaction was conducted in a volume of 400 μl, produced by adding 306 μl of H_2_O and 2 μl of a DNA template (LT1 or LT2, 500 μM) to the phosphorylation reaction mixture above. After heating at 90°C for 3 min and cooling down at room temperature (RT) for 10 min, 40 μl of 10× T4 DNA ligase buffer (400 mM Tris–HCl, 100 mM MgCl_2_, 100 mM DTT, 5 mM ATP, pH 7.8 at 25°C) and 2 μl of T4 DNA ligase (5 U/μl) were added. This mixture was incubated at RT for 2 h before heating at 90°C for 5 min to deactivate the ligase. The ligated circular DNA molecules were concentrated by standard ethanol precipitation and purified by 10% dPAGE (Supplementary Figure S1). The concentration of the circular DNA template was determined spectroscopically.

### *In vitro* selection of circular aptamers

In the first round, the circular DNA library (1 nmol), which was denatured at 95°C for 10 min and then cooled on ice for 15 min, was incubated with beads (1 × 10^8^ beads / mL) with rotation for 1 h at room temperature in 100 μl of binding buffer (1× PBSMT, pH 7.2) containing 137 mM NaCl, 2.68 mM KCl, 8.1 mM Na_2_HPO_4_, 1.76 mM KH_2_PO_4_, 1 mM MgCl_2_, and 0.025% Tween-20. The tube was then applied to a magnet (Qiagen), the supernatant was collected into a new tube. The collected circular oligonucleotides were then incubated with bead-bound thrombin (13.5 pmol) with rotation for 1 h at room temperature in 100 μl of 1× PBSMT. The tube was then applied to a magnet, the supernatant was removed, and the beads were washed three times with 1× PBSMT (100 μl). Bound aptamers were eluted with 50 μl of ultrapure water at 95°C for 15 min with the help of magnet. After recovery by ethanol precipitation, the circular DNA denoted circular thrombin binding aptamer (CTBA) was amplified by two rounds of RCA, restrict digestion and circularization. The RCA for the first round of selection was performed in 50 μl of 1× RCA buffer (made from 10× stock, which is made of 330 mM Tris-acetate, pH 7.9 at 37°C, 100 mM magnesium acetate, 660 mM potassium acetate, 1% (v/v) Tween 20, 10 mM DTT) containing CTBA derived from last step, 2 μM LT1 (100 pmol), 1 mM dNTP. After heating at 90°C for 3 min, the solution was cooled at room temperature for 10 min. Subsequently, 0.5 μl of phi29 DNA polymerase (10 U/μl) was added, followed by incubation at 30°C for 30 min. Finally, the mixture was heated to 65°C for 10 min to deactivate the polymerase. To the RCA reaction mixture above, 2 μl of 500 μM LT2 (1 nmol) was introduced. The mixture was heated at 90°C for 3 min and cooled at RT for 10 min, followed by the addition of 10 μl of 10× Fast Digestion Buffer (100 mM Tris–HCl, pH 8.0, 50 mM MgCl_2_, 1 M NaCl, 1 mg/ml BSA) and 5 μl of FastDigest EcoRV (unit size 400 reactions; the total volume is 400 μl). The total reaction volume was increased to 100 μl. The reaction mixture was then incubated at 37°C for 16 h (29). The restriction enzyme was inactivated at 65°C for 10 min. The monomerized RCA products were concentrated by standard ethanol precipitation and purified by dPAGE. The DNA was then eluted and circularized into circular DNA template B (CDTB), which was used for the second RCA reaction. The reaction condition was identical to the first RCA except for the replacement of LT1 with LT2. For the restriction digestion after RCA, LT2 was replaced LT1. Serum was introduced into the binding buffer after two rounds of selection. Seven rounds of selection were conducted while the amount of the serum was increased from 2% (round 3) to 5% (round 4), 10% (round 5), 20% (round 6) and 50% (round 7). The DNA pool from round 7 was used for deep sequencing.

### Sequencing protocol

CTBA in round 7 was digested into linear DNA sequences as previously described. 2 μl of 0.05 μM linear CTBA was amplified by PCR. There were two PCR steps. In PCR1, a reaction mixture (50 μl) was prepared to contain the DNA above, 0.4 μM each of forward primer (FP) and reverse primer (RP; their sequences are provided in Supplementary Table S1), 200 μM each of dNTPs (dATP, dCTP, dGTP and dTTP), 1× PCR buffer (75 mM Tris–HCl, pH 9.0, 2 mM MgCl_2_, 50 mM KCl, 20 mM (NH_4_)_2_SO_4_) and 1.5 U Taq DNA polymerase. The DNA was amplified using the following thermocycling steps: 94°C for 3 min; 15 cycles at 94°C (30 s), 42°C (45 s) and 72°C (45 s); 72°C for 1 min. 1 μl of the PCR1 product was diluted with H_2_O to 100 μl, 2 μl of which was used as the template for PCR2 using the same primers (their sequences are provided in Supplementary Table S1) while following the same protocol above for PCR1 except that the annealing temperature increased to 48°C. The DNA product generated in PCR2 was analyzed and purified by 2% agarose gel electrophoresis and sent out for deep sequencing. Paired-end next generation sequencing (NGS) was done using an Illumina Miseq system at the Novogene Facility. Forward and reverse reads were sorted by tag and exported as FASTQ files using the Illumina Basespace platform. Primer domains were removed and paired-end reads were merged using PANDAseq 2.6, only sequences possessing perfect complementarity between paired-end reads were output in FASTA format for further analysis (30). Sequences were duplicated and tagged with copy number using USEARCH v7.0.1090_i86linux32 sequence analysis package (31). USEARCH was also used for clustering of duplicated populations using the cluster_smallmem command at 0.9 identity threshold. PANDAseq and USEARCH software packages were run on Ubuntu Linux 12.04 LTS. Analysis of sequence populations, rankings and base composition were done using Microsoft Excel 2010 running on a Windows 8 PC.

### Thrombin inhibition assays

Thrombin inhibition assays were performed by monitoring the formation of insoluble fibrin by measuring the absorbance of the reaction at 350 nm on a microplate reader (Tecan) at 25°C. To determine the half-maximal inhibitory concentrations (IC_50_) of the thrombin inhibitors, we prepared reactions containing 0.1 nM thrombin, 2 μM fibrinogen (Sigma) and various inhibitors at a range of different concentrations in 100 μl of selection buffer in duplicate. For each thrombin inhibitor, the absorbance at steady state were blank subtracted then normalized. These values were fitted to a four-parameter logistic model (Origin) by nonlinear regression to calculate the IC_50_. The model takes the form: *y* = *D* + (*A* – *D*)/(1 + (*x*/*C*)^*B*^, where *y* = normalized absorbance, *x* = the inhibitor concentration, and with the four parameters, *A* = absorbance with no inhibition, *B* = the slope factor, *C* = IC_50_ and *D* = absorbance at maximum inhibition (32).

### Circular dichroism analysis

Circular dichroism (CD) studies were carried out using a JASCO J-600 instrument according to the manufacturer's instructions. All constructs were scanned from 320 to 220 nm at 20 μM DNA in a 0.1-cm quartz cuvette. All DNA samples were incubated for 30 min at room temperature in the 1× selection buffer before scanning.

### Fluorescence measurements of G-quadruplex–ligand interaction

Thioflavin T (ThT; 1 μM) was incubated with a relevant circular DNA (1 μM) in binding buffer for 10 min. Fluorescence measurements were carried out in a 96-well assay plate using a microplate reader (Tecan infinite M1000 Pro, Männedorf, Switzerland) under room temperature. The excitation wavelength of the solution was set at 425 nm, and the emission spectra were collected from 450 to 600 nm with a step of 2 nm.

### Electrophoretic mobility shift assays (EMSA)

Binding reactions were performed in 20 μl of 1× binding buffer containing <50 pM ^32^P-labeled CTBA4T (or TBA_15_) and various concentrations of thrombin (0-32 nM or 0–1000 nM). After incubation for 30 min, the reaction mixtures were spiked with 6× loading buffer (6× = 15% Ficol, 1× binding buffer), and then loaded into the wells of a non-denaturing 0.5× TBE, 10 mM KCl polyacrylamide gel (10%) at 4°C. Visualization of DNA bands was achieved by storage phosphor screen and imaged on a GE Typhoon Biomolecular Imager and the resulting bands were quantified using ImageJ software.

### Methylation interference assay

A solution of 10 μM FAM-labeled DNA (500 pmol) in 1× PBSMT was heated to 90°C for 1 min and cooled to room temperature. For control samples, the same amount of FAM-labeled DNA in ddH_2_O was heated to 90°C for 1 min and cooled to room temperature quickly to disrupt the folding. The methylation reaction was initiated by adding an equal volume of 0.4% (v/v) DMS (freshly made) to each of the above reaction mixture, followed by incubation at room temperature for 40 min. Methylated DNA was recovered by ethanol precipitation, followed by two washes in 70% ethanol, and subjected to methylation-dependent cleavage in 50 μl 10% (v/v) piperidine for 30 min at 90°C. The resultant cleavage products were dried under vacuum and analyzed by 20% denaturing PAGE.

### Competitive binding assay

Binding reactions were prepared with ^32^P-labeled CTBA4T at 500 pM, 50 pM thrombin and a titration of unlabeled competitor aptamer (Published thrombin aptamers TBA_15_ (Bock *et al.*) and ‘TBA_29_’ (Tasset *et al.*) targeting fibrinogen and heparin epitopes respectively were used as competitors of CTBA4T for thrombin binding). Competition reactions were performed in 1× binding buffer and incubated at room temperature for 1 h. Samples were prepared for electrophoresis by addition of loading dye to 1× final concentration.10% 0.5× TBE native PAGE was prerun at 200 V for 1 h at 4°C. Samples were loaded and gel was run at 200 V for 1 h. Storage phosphor screens were exposed for 1 h. Visualization of DNA bands was done using a GE Typhoon Biomolecular Imager and the resulting bands were quantified with ImageJ software. Non-linear regression performed in GraphPad Prism using a one-site competitive binding model to estimate IC_50_. *Y* = (top –bottom)/(1+10^(*X* – log IC50)^) + bottom.

### Thermal melting temperature analysis

The high resolution melting experiments were carried out with Roche LightCycler96 quantitative fluorescence PCR instrument. Samples were prepared at a concentration of 10 μM in LightCycler^®^ 480 high resolution melting master, which contained ResoLight Dye, a double-stranded DNA binding Dye. The high resolution melting curves were obtained by following the fluorescence change in the 37–90°C range using a scan rate of 0.05°C/s and 20 readings/°C.

### Stability assay

10 μM FAM-labeled anti-thrombin aptamers (1000 pmol) were incubated in 50% human serum at 37°C. At established times, ranging from 0 to 24 h, 10 μl of samples were collected, heated at 90°C for 10 min and stored at –20°C for further analysis. The samples were then mixed with 10 μl 2× denaturing gel loading buffer and analyzed for 10% denaturing PAGE. Visualization of the gel was done using a Bio-Rad image system and the bands were quantified with ImageQuant software.

### Prothrombin time assay

Prothrombin time (PT) assay on human plasma samples was measured by using a semi-automatic coagulometer with a specific kit for blood coagulation test (Sun Biotech, Shanghai, China). Briefly, this method relies on the high sensitivity of thromboplastin reagent based on recombinant human tissue factors. The addition of recombiplastin to the plasma, in presence of calcium ions, initiates the activation of extrinsic pathway converting the fibrinogen into fibrin, with a formation of solid gel. The procedure was performed according to the manufacturer's instructions. In our experimental conditions, each thrombin inhibitor (200 μM, 1.7 μl) was incubated with 100 μl of plasma at 37°C for 3 min, after that 200 μl of the kit solution containing recombiplastin was added with consequent activation of extrinsic pathway. The PT measurement, for each incubation time, was produced in triplicate and the average and its standard error values were calculated and expressed as seconds. The basal clotting time was determined by measuring the clotting time in absence of any thrombin inhibitor.

## RESULTS

### Directed evolution of circular aptamers in serum

**Figure 1.**
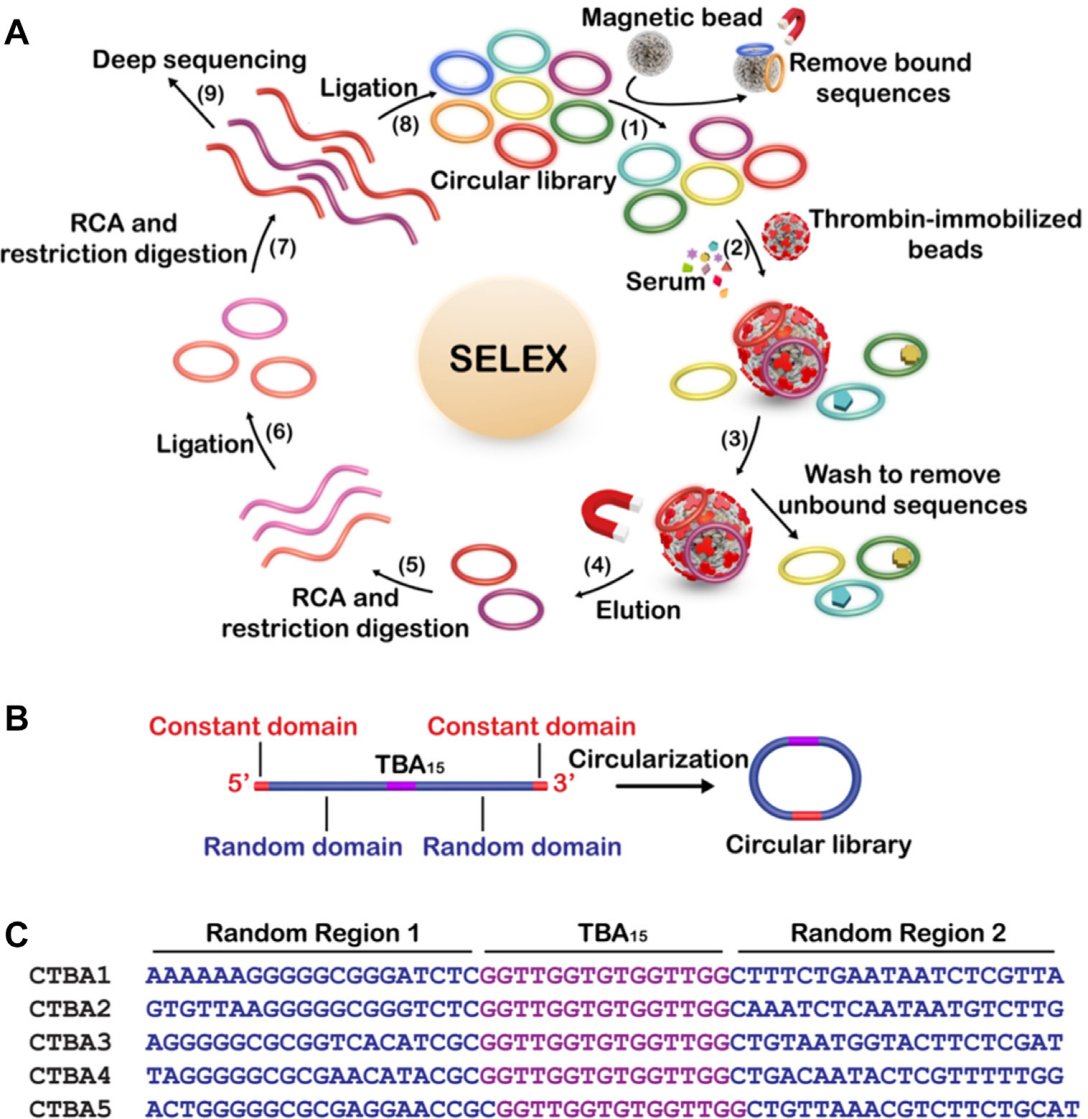
(**A**) Strategy for adapting TBA_15_ into a circular DNA aptamer in serum. (**B**) Circular DNA library design. (**C**) The sequence of top 5 aptamer candidates. Each circular aptamer also contains the following constant sequence of 5′-TGTCT CGGAT ATCTC GACTA GTCA-3′.

The adaption strategy is illustrated in Figure 1A. The circular DNA library was prepared by T4 DNA ligase mediated circularization of Lib, a linear DNA library containing TBA_15_ flanked with a 20-nt random region and a constant region on each side (see Figure 1B for its construction, and Supplementary Table S1 in the Supporting Information for the sequences of all DNA molecules used in this work). The yield of circularization was calculated to be 86% (Supplementary Figure S1). The use of two random regions to flank TBA_15_ was intended to provide TBA_15_ the best chance to recruit additional functional nucleotides from both ends as well as minimize the impact of cyclization on the binding activity of TBA_15_. The circular DNA pool was first mixed with bare carboxylic acid magnetic beads (CAMB) in a counter-selection step to remove bead-binding sequences (step 1). The unbound DNA was incubated with the CAMB coated with thrombin (step 2). This step was conducted directly in serum. The unbound DNA molecules were removed by washing (step 3) and the bound species were eluted by heating (step 4). The DNA recovered was then amplified by a circle-to-circle amplification strategy we reported before (29). Briefly, the bound circular DNA from step 4 was amplified by rolling circle amplification (RCA) (33–37). Following RCA and restriction digestion of long RCA products into monomers (step 5), circularization was performed to produce a new circular DNA (step 6), which was used as the template for another RCA and restriction digestion (step 7). Circularization was again performed to produce the circular DNA pool for the next round of selection (step 8). It is noteworthy that the circle-to-circle amplification has never been attempted in previous aptamer selection experiments. It represents a simpler method over polymerase chain reaction (PCR) because it is isothermal and equipment-free.

### Selected circular aptamer effectively inhibits thrombin

∼10^14^ distinct circular DNA molecules were used as the initial DNA library, with which seven rounds of selection were carried out under increasing stringency to favor isolation of circular aptamers with the best activity in serum (2%, 5%, 10%, 20% and 50% serum in rounds 3, 4, 5, 6 and 7, respectively). The DNA pool from round 7 was subjected to high-throughput DNA sequencing (Supplementary Table S2). 4 015 918 sequence reads were obtained; the top five sequences account for ∼30%, indicating effective enrichment (the top fifty sequences from deep sequencing result were shown in Supplementary Figure S2).

**Figure 2.**
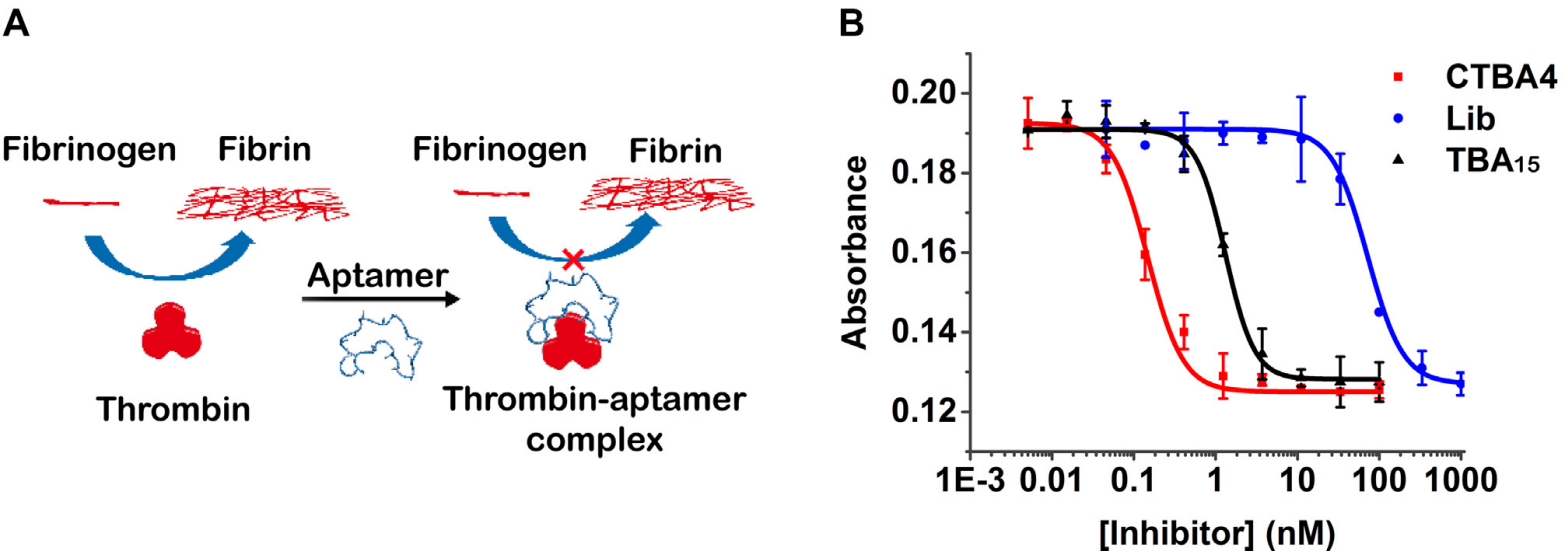
(**A**) Inhibition of thrombin-induced fibrin polymerization (TIFP) by an aptamer. (**B**) Inhibition of TIFP by CTBA4, TBA_15_ and Lib, measured as absorbance at 350 nm as the function of inhibitor concentration. The data were fitted to a four-parameter logistic model.

We assessed the top five sequences for their ability to inhibit thrombin-induced fibrin polymerization (TIFP), a process closely associated with blood-clotting (Figure 2A). Each circular aptamer was incubated with thrombin and fibrinogen. By monitoring A_350_ (absorbance at 350 nm; Supplementary Figure S3), we can determine the amount of insoluble fibrin produced and calculate the half maximal inhibitory concentration, IC_50_ (Supplementary Table S3). CTBA4 showed the best activity (Figure 2B), with an IC_50_ of 0.15 nM. As controls, TBA_15_ and the original circular DNA library had the IC_50_ values of 1.31 and 72.14 nM, respectively. The TIFP inhibition efficiency of CTBA4 is 480-fold better than the circular library and 9-fold better than TBA_15_. These results are consistent with our speculation that in vitro selection can produce highly functional circular aptamers from a linear aptamer.

### Aptamer characterization and optimization

**Figure 3.**
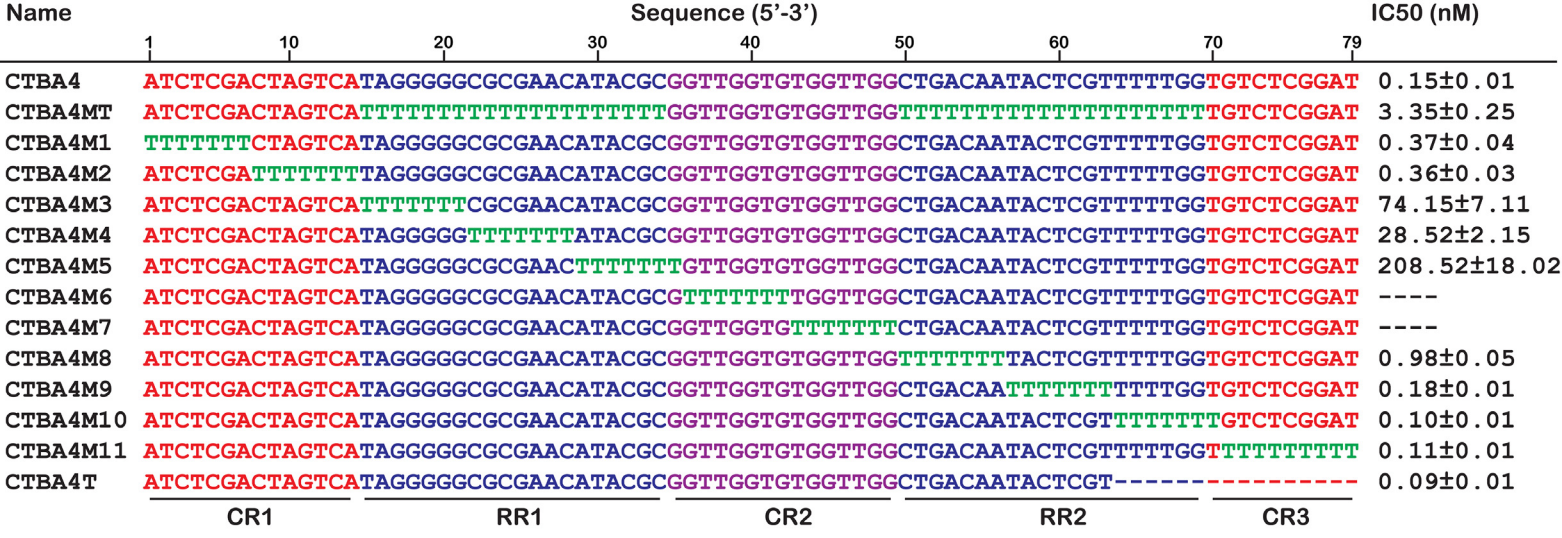
T-substitution study of CTBA4.

The 79-nt sequence of CTBA4 can be divided into 5 sequence regions as shown in Figure 3: CR1 (constant region 1, A_1_–A_14_), RR1 (random region 1, T_15_–G_34_), CR2 (G_35_–G_49_), RR2 (C_50_–G_69_), CR3 (T_70_–T_79_). A mutant aptamer of CTBA4, named CTBA4MT, in which all the nucleotides in the selected RR1 and RR2 sequence elements were mutated to dT-nucleotides, was made and tested using TIFP. The IC_50_ value of CTBA4MT increases by 22-fold, suggesting that some or all of the nucleotides within the RR1 and RR2 elements in CTBA4 were indeed functionally important. We then carried out T-tract walking experiment to determine the importance of a group of consecutive nucleotides within each region of CTBA4; in this experiment, each chosen nucleotide group was substituted with the same number of T residues. Substitutions within CR2 led to the complete loss of activity (CTBA4M6 and CTBA4M7), this was not surprising because CR2 was the seeding aptamer. Interestingly, substitutions within RR1 resulted in significantly increased IC_50_ (IC_50_ of CTBA4M3, CTBA4M4 and CTBA4M5 increased by 494-, 190- and 1390-fold, respectively), indicating nucleotides in this region were evolved to play important roles in thrombin binding. Substitutions elsewhere had no or less significant impact on IC_50_, suggesting most residues within CR1, RR2 and CR3 are unimportant for thrombin recognition. Among these mutants, CTBA4M10 and CTBA4M11 exhibited nearly identical IC_50_ to the mother sequence. Based on these observations, we synthesized a shortened aptamer, CTBA4T, in which T_65_-T_79_ was deleted. This molecule had an IC_50_ of 90 pM.

It was worth noting that there are five consecutive G residues in RR1 of CTBA4. In fact, the existence of ∼5 consecutive guanines in either RR1 or RR2 is a common feature of all top 50 selected sequences (green Gs in Supplementary Figure S2; 45′5Gs, 3′6Gs, 1′7Gs and 1′4Gs), suggesting an important structural or functional role for this new evolved element (more to follow).

### CTBA4 forms a unique G-quadruplex structure

**Figure 4.**
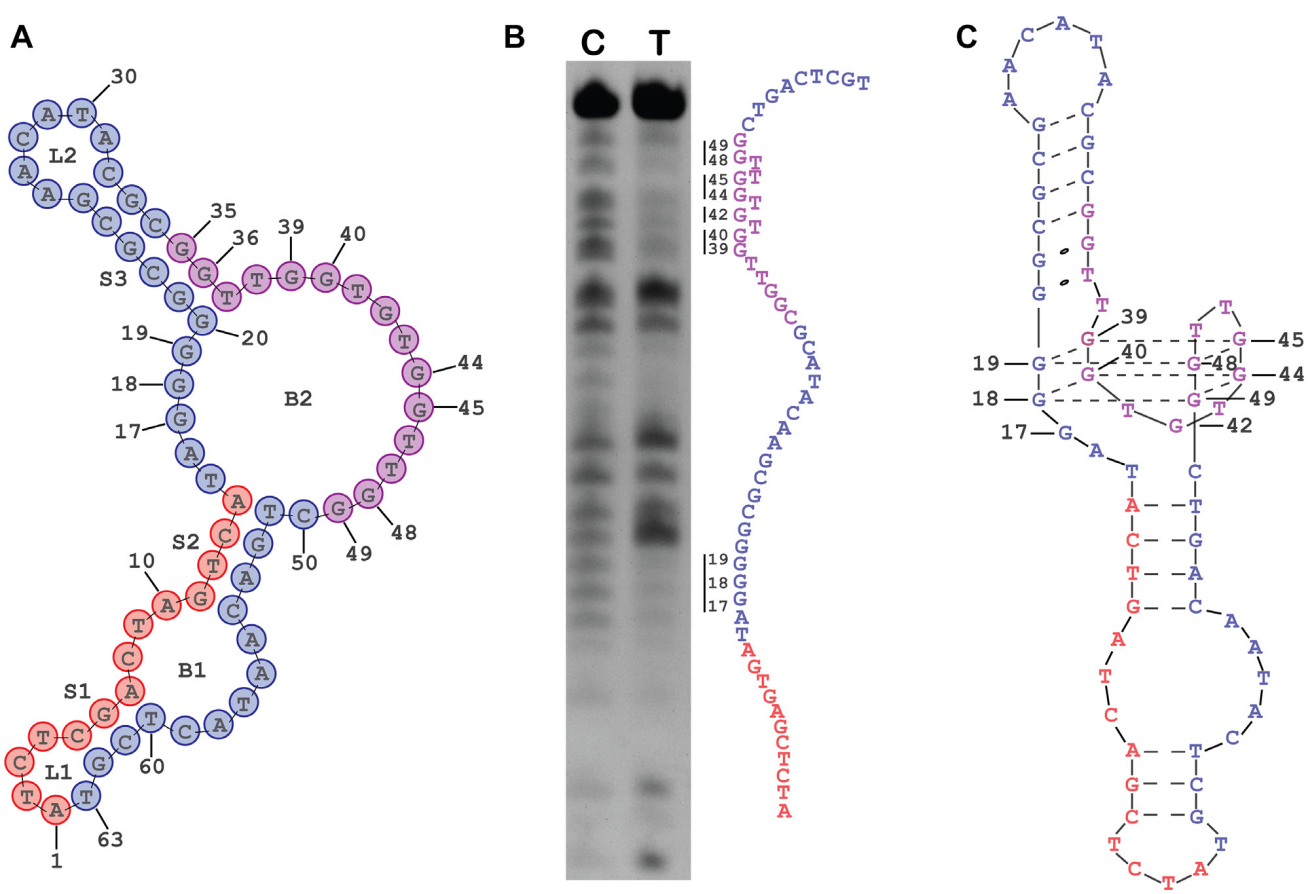
(**A**) Predicted secondary structure of CTBA4T. (**B**) Methylation interference of LTBA4T-B1: 20% dPAGE of LTBA4T-B1 after methylation interference and piperidine cleavage. Four pairs of guanines showing significant methylation interference are highlighted along with their base number. C refers to the control sample (without thrombin) and T refers to the test sample (with thrombin). (**C**) The proposed structure for CTBA4T.

To understand the structural basis of CTBA4T for significantly increased TIFP inhibition efficiency over TBA_15_, we used mfold to predict Watson-Crick elements within CTBA4T. The structure with the lowest free energy is shown in Figure 4A. It contains two loops (L1, L2), two bulges (B1, B2) and three stems (S1, S2, S3). One interesting prediction is the placement of G_35_G_36_T_37_ in S3, as these 3 nucleotides were supposed to fold into the G-quadruplex (G4) structure for thrombin binding in the original library design. Since the remaining nucleotides in the original seeding aptamer are still predicted to be a single-stranded element as part of B2 and there are also consecutive G residues in the other single-stranded element of B2, we were wondering if a new G4 structure is formed that is now responsive for thrombin binding.

The technique of circular dichroism (CD) was employed to provide evidence for the existence of G4 in the structure of CTBA4T (Supplementary Figure S4). The CD spectrum indeed contains a negative peak close to 260 nm and a positive peak close to 290 nm, features that are indicative of an antiparallel G-quadruplex (38). The slight shifting of the two peaks toward wavelengths lower than those usually seen with pure antiparallel G4 structures may be caused by the influence of the flanking duplex structures (39,40).

Methylation interference was performed to identify guanine residues involved in the G4 formation; this technique can detect the sensitivity of N7 atoms of guanines to methylation and is widely used for G4 structure probing (41,42). Because it is difficult to determine methylation interference directly with a circular DNA molecule, we sought to use the linear counterpart (LTBA4T). However, a comparison of the predicted secondary structures for LTBA4T (Supplementary Figure S5b) and CTBA4T reveals significant disparities. However, a slightly modified version of the aptamer, CTBA4T-B1 was predicted to form a structure (Supplementary Figure S5c) comparable to CTBA4T; for this reason, its linear form, LTBA4T-B1 (Supplementary Figure S5d) was used for the methylation interference experiment.

Ten guanines within B2 exhibited significant methylation interference (Figure 4B), and they can be grouped as four pairs of two consecutive G residues: G_18_G_19_ (or G_17_G_18_), G_39_G_40_, G_44_G_45_, G_48_G_49_. This points to a possibility that these guanines form the two-tiered G4 as illustrated in Figure 4C. G_17_ (or G_19_) and G_42_ also showed strong methylation interference. It is possible that these two guanine residues participate in some important tertiary interactions that involve their N7 atoms. Alternatively, being located very close to the G4 element may place them in tight spatial arrangements, preventing them from tolerating the bulky methyl group on N7. It is noteworthy that G_35_ and G_36_ did not exhibit substantial methylation interference, consistent with the structural model predicted with mfold in which these two G nucleotides are arranged as part of the S3 stem (the N7 atoms of guanine in DNA duplex does not produce strong methylation interference).

The methylation experiment strongly suggests that G_18_G_19_ or (G_17_G_18_) within RR1 was evolved to partner with G_39_G_40_, G_44_G_45_, and G_48_G_49_ within CR2 (i.e. the seeding aptamer region) to create a new G4 structure. In the process, the G_35_G_36_ pair that are an integral part of the G4 structure of TBA_15_ were re-arranged into a stem that is next to the G4 element (Figure 4C). Since G_17_G_18_G_19_ all showed methylation interference and they can be arranged as G_18_G_19_ pair or as G_17_G_18_ pair, for the formation of a G4 structure, an alternative structure in which G_17_G_18_ was used to create the G4 pairing is shown in Supplementary Figure S6.

The proposed structure is very unique in that it contains both G-quadruplex and duplex elements. This structure differs from all known G4 structures in two major ways (43–46). First, it has a circular topology as common G4 structures are made of linear nucleic acids. Second, it has two large and structured loop elements. This may be also linked to its circular topology, because, as a circular molecule, CTBA4T may require more nucleotides to build inter-strand connecting loops to stabilize its core G4 element. Given its better performance over TBA_15_, the isolation of CTBA4T points to the added advantage of evolving aptamers from circular DNA libraries since this approach can generate aptamers with structural folds that are unique to the circular topology.

There are also many unpaired nucleotides near and away from the G-quadruplex element, which may further participate in some very important tertiary interactions that ultimately contribute to the significantly increased TIFP inhibition efficiency and affinity over TBA_15_.

**Figure 5.**
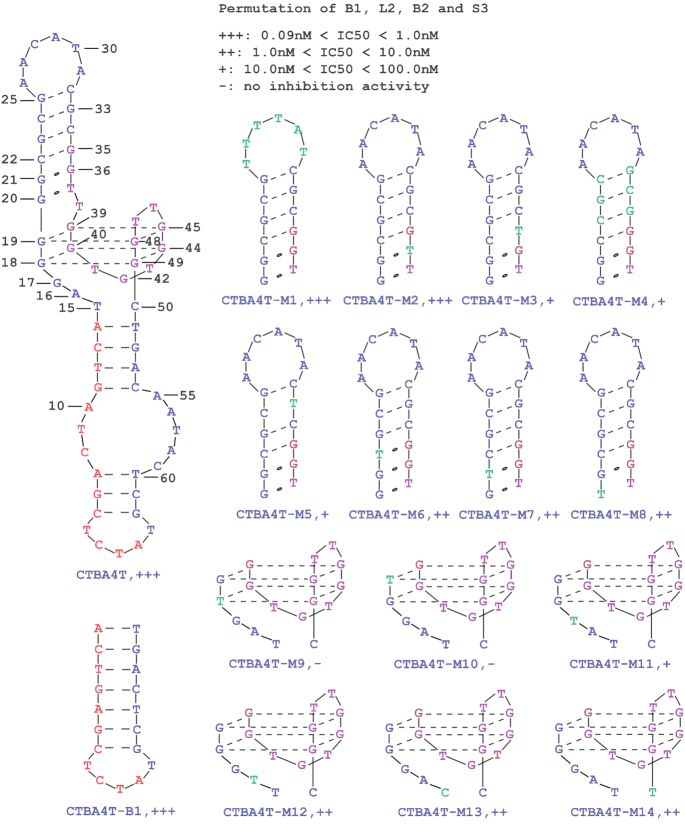
Permutations of B1, B2, S3 and L2. The putative secondary structure of CTBA4T is shown on the left, and mutant aptamers are shown on the right. The name of each mutant aptamer is given underneath each altered motif. Nucleotides shown in green are the actual altered nucleotides in each construct in comparison to CTBA4T.

We next designed 14 mutants of CTBA4T with mutations targeting B1, L2, B2, S3 and examined their relative TIFP inhibition efficiencies in an attempt to gather information on the functionality of these structural elements (Figure 5). We found that B1 could be eliminated altogether and the resultant mutant CTBA4T-B1 retained the full TIFP inhibition activity. Similarly, the full activity was maintained when the sequence of L2 was scrambled (CTBA4T-M1). These two observations indicate that B1 and L2 do not play any role in target recognition.

Several mutants (CTBA4T-M2-8) were designed to examine functionalities of the nucleotides in S3. The most interesting observation is that the G-toT mutation of G_36_ (CTBAT-M2), which is known to participate in the formation of G4 in the seed aptamer TBA_15_, did not affect the TIFP inhibition efficiency. This finding is consistent with our proposed structural model of CTBA4T that does not involve G_36_ in the G4 structure formation. The TIFP inhibition activities observed with the other mutants made in S3 largely support the existence of the proposed S3 stem element, but also suggest that nucleotides within this element may play important, but not absolutely essential, roles in the structural formation and/or target recognition of the new evolved aptamer.

Six mutants (CTBA4T-M9-14) targeting B2 were also tested. The G-toT mutation of G_18_ (CTBA4T-M9) and G_19_ (CTBA4T-M10) led to complete loss of TIFP inhibition activity, consistent with the proposed structural model of CTBA4T that places G_18_ and G_19_ as part of the G4 element. The G-toT mutant of G_17_ (CTBA4T-M11) resulted in signitifcant loss of TIFP inhibition activity while the mutants carrying T15C, A16T, or C50T mutation (CTBA4T-M12-14) exhibited substantial activity losses. These observations suggest that these four unpaired nucleotides next to the proposed G4 structure may participate in target recognition or play important structural roles.

The existence of a G4 structure in CTBA4 was further confirmed with the experiment with Thioflavin T, a fluorescent dye that specifically binds G4 and produce enhanced fluorescence (Supplementary Figure S7) (47).

### High binding affinity and thermal stability

CTBA4T and CTBA4T-B1 were then radioactively labeled and tested for thrombin binding by electrophoretic mobility shift assays (EMSA; Supplementary Figure S8). The fraction of bound aptamer (top band) was plotted as a function of thrombin concentration, resulting in a *K*_d_ of 23 pM and 19 pM for CTBA4T and CTBA4T-B1, respectively. Strikingly, their binding affinity was much better than the circular control, CTBA4MT, and their linear forms (Supplementary Figure S9), showing a huge improvement from their mother sequence TBA_15_ (∼800-fold for CTBA4T and ∼1000-fold for CTBA4T-B1).

It is noteworthy that CTBA4T recognizes the same epitope in thrombin, exosite I, that is recognized by TBA_15_. This was clear from the competitive binding assay shown Supplementary Figure S10: CTBA4T in the aptamer/thrombin complex can be replaced by TBA_15_ at high concentrations. This is not surprising as TBA_15_ was used as the seeding aptamer. As a control, thrombin-complexed CTBA4T could not be displaced by TBA_29_, a 29-nt DNA aptamer that is known to bind exosite II of thrombin (Supplementary Figure S9) (48).

Thermal melting experiments were carried out to determine the relative thermal stabilities for the circular aptamers. The thermal melting temperatures (T_m_) obtained from high resolution melting curves were shown in Supplementary Table S4. Inspection of Supplementary Table S4 reveals a remarkable enhancement (∼+10°C) of the thermal stability for CTBA4T and CTBA4T-B1 versus their linear form (LTBA4T and LTBA4T-B1).

### Nuclease resistance in human serum and the anticoagulant activity in blood

**Figure 6.**
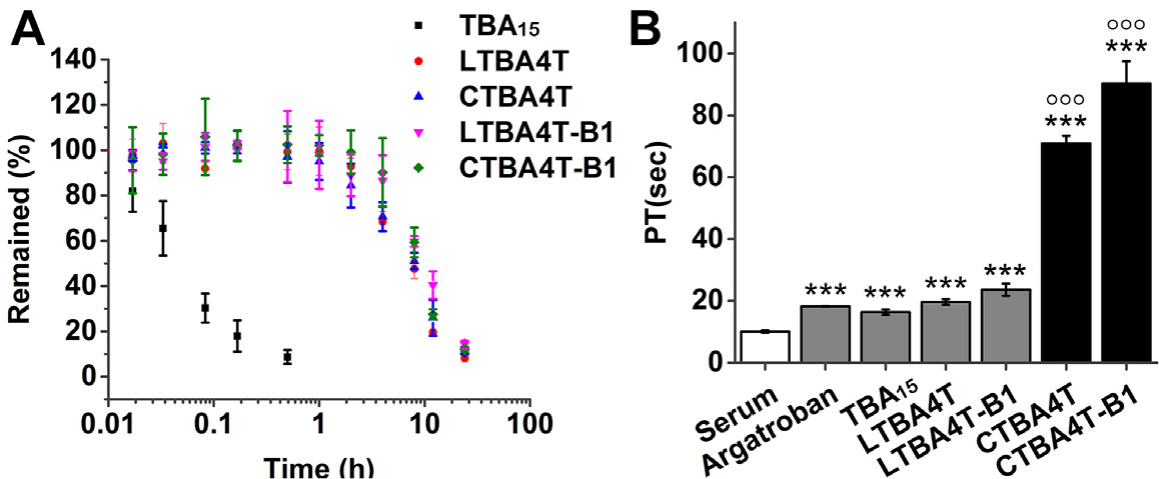
(**A**) Stability of TBA_15_, LTBA4T, CTBA4T, LTBA4T-B1 and CTBA4T-B1 in serum. (**B**) PT values of Argatroban, TBA_15_, LTBA4T, LTBA4T-B1, CTBA4T and CTBA4T-B1. ***P < 0.001 versus vehicle, °°°*P* < 0.001 versus TBA_15_ (*n* = 3).

The nuclease resistance of CTBA4T, CTBA4T-B1 and their linear forms in biological media was investigated next. Figure 6A shows that the half-life of CTBA4T, CTBA4T-B1 and their linear forms in 50% human serum was circa 8 h, while the half-life of TBA_15_ was less than 5 min, illustrating the superb capability of the selected aptamers against nuclease degradation. To further evaluate the anticoagulant activity in blood, CTBA4T, CTBA4T-B1, LTBA4T, LTBA4T-B1, TBA_15_ and Argatroban (a small molecule inhibitor of thrombin) were subjected to Prothrombin Time (PT) assay (49,50). CTBA4T and CTBA4T-B1 showed significantly prolonged PT over LTBA4T, LTBA4T-B1, TBA_15_ and Argatroban (Agtb), while CTBA4T-B1 exhibited the highest PT value, indicating a much higher anticoagulant activity in human plasma (Figure 6B). Interestingly, despite that LTBA4T and LTBA4T-B1 are as stable as CTBA4T and CTBA4T-B1 in human serum, they produced much poorer PT values. Given the observation that each circular form exhibits a higher melting temperature than their linear counterpart, we believe the high PT value of each circular construct reflects their high structural stability and thus their ability to resist the interference from other DNA binding molecules in biological media. The result clearly underscores the potential therapeutic value of CTBA4T-B1 for blood coagulation.

## DISCUSSION

In summary, we report the first effort to derive a protein-binding circular DNA aptamer directly in serum, resulting in the isolation of a highly functional circular DNA aptamer that specifically and strongly binds thrombin, and shows potent activity to inhibit thrombin-induced fibrin polymerization. This condition-driven strategy to evolve a functional circular aptamer from a precursor linear aptamer requires no structural information for the target protein, and thus can be expanded to any protein target with and without determined structures. The isolation of CTBA4T-B1 also highlights the advantage of selecting circular aptamers directly in biological media, because this approach can produce aptamers with improved physical stability and functionality in the intended biological media. The use of RCA as an amplification tool has also significantly simplified the enrichment of circular aptamers. Taken together, we envision that the demonstrated strategy can be exploited for creating a wide variety of high-quality circular DNA aptamers that are both stable and highly functional in real biological samples, thus boosting the practical utilities of DNA aptamers for biomedical applications.

## Supplementary Material

gkaa800_Supplemental_File
