# Supplementary material for: Evolution of a highly functional circular DNA aptamer in serum
Source: Nucleic Acids Res. 2020 Oct 6;48(19):10680–90. doi: 10.1093/nar/gkaa800 (PMC7641760; doi:10.1093/nar/gkaa800)
Supplement: gkaa800_Supplemental_File [file gkaa800_supplemental_file.pdf]

Supporting Information

Evolution of a highly functional circular DNA aptamer in serum

Yu Mao, Jimmy Gu, Dingran Chang, Lei Wang, Lili Yao, Qihui Ma, Zhaofeng Luo, Hao Qu, Yingfu Li* and Lei Zheng*

Supplementary Figures

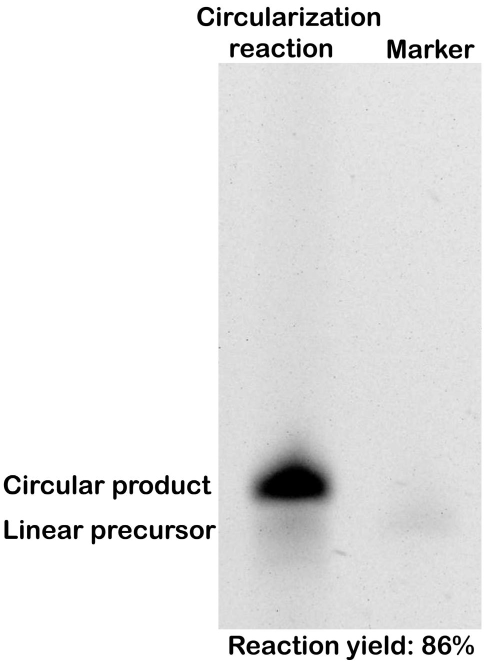

Figure S1. A 10% denaturing PAGE for the separation of the ligated circular DNA from the linear DNA precursor. The yield of circularization was calculated to be 86%. The marker was the linear library before circularization.

Name	Sequence (5'-3')	Multiplicity	% of Total
CTBA1	ATCTCGACTAGTCAAAAAAAGGGGGCGGGATCTCGGTTGGTGTGGTTGGCTTTCTGAATAATCTCGTTATGTCTCGGAT	338573	8.43
CTBA2	ATCTCGACTAGTCAGTGTTAAGGGGGCGGGTCTCGGTTGGTGTGGTTGGCAAATCTCAATAATGTCTTGTGTCTCGGAT	278871	6.94
CTBA3	ATCTCGACTAGTCAAGGGGGCGCGGTCACATCGCGGTTGGTGTGGTTGGCTGTAATGGTACTTCTCGATTGTCTCGGAT	278053	6.92
CTBA4	ATCTCGACTAGTCATAGGGGGCGCGAACATACGCGGTTGGTGTGGTTGGCTGACAATACTCGTTTTTGGTGTCTCGGAT	226305	5.64
CTBA5	ATCTCGACTAGTCAACTGGGGGCGCGAGGAACCGCGGTTGGTGTGGTTGGCTGTTAAACGTCTTCTGCATTGTCTCGGAT	111078	2.77
CTBA6	ATCTCGACTAGTCAAATTAATGGGGCGATTCAACGGTTGGTGTGGTTGGGATTTGTTCGTCGGTAACAGTGTCTCGGAT	80153	2.00
CTBA7	ATCTCGACTAGTCAATATAAAAGAGGGGGGTCAAGGTTGGTGTGGTTGGCAGTTTTGTCTTTAGTCTCGTGTCTCGGAT	71113	1.77
CTBA8	ATCTCGACTAGTCAAAACGAAGGGGGCGGAATCTCGGTTGGTGTGGTTGGCTGTATTGCTTATTCAAATGTGTCTCGGAT	70124	1.75
CTBA9	ATCTCGACTAGTCAAAATGAGGGGGCAGTTTCGTGGTTGGTGTGGTTGGCGGTATGCAGGTCTGTCTCGTGTCTCGGAT	65984	1.64
CTBA10	ATCTCGACTAGTCACTGGAGGGGGCGGCTCTCTCGGTTGGTGTGGTTGGCAAATTGGCTGTCTAAAATATGTCTCGGAT	60808	1.51
CTBA11	ATCTCGACTAGTCATTAAAGGGGGCAGGTCTCGTGGTTGGTGTGGTTGGCTACATGCAGGTCTCGTCTTTGTCTCGGAT	56738	1.41
CTBA12	ATCTCGACTAGTCACAGAAATTATTAAATGTCTCGGTTGGTGTGGTTGGCAATATAGGGGGCGGATCATTGTCTCGGAT	54560	1.36
CTBA13	ATCTCGACTAGTCAGTATGCAGAAATGCTAAATGTGGTTGGTGTGGTTGGCTTGGGGGCGCAAAAAGTTTTGTCTCGGAT	54226	1.35
CTBA14	ATCTCGACTAGTCATGGGCAAGGGGGCGTATCGCGGTTGGTGTGGTTGGCTGATAATTACTCCTCGTTATGTCTCGGAT	53175	1.32
CTBA15	ATCTCGACTAGTCAATAAATCTTTAGGGGGCTAAGGTTGGTGTGGTTGGCAAAGTTCTAAGCAAGTCTCTGTCTCGGAT	41018	1.02
CTBA16	ATCTCGACTAGTCAAAAGGAAAAGGATTGGTAATGGTTGGTGTGGTTGGAAAAAGACTCTCGGATCTTCTGTCTCGGAT	39686	0.99
CTBA17	ATCTCGACTAGTCAATGTATGGGGGCGGATTTTGCGGTTGGTGTGGTTGGCTCAATGAATTTCGTTCTCGTGTCTCGGAT	38565	0.96
CTBA18	ATCTCGACTAGTCAAGAAATGGGGGCGTCTGTTGCGGTTGGTGTGGTTGGCGTTCTAAAGTTTTAATCTCTGTCTCGGAT	37098	0.92
CTBA19	ATCTCGACTAGTCAGTCAAAATAGATAAAATAACGGTTGGTGTGGTTGGTAATGGGGCGAATGCTATGTTGTCTCGGAT	36981	0.92
CTBA20	ATCTCGACTAGTCAAAGTAGGGGGCGGTATCTGCGGTTGGTGTGGTTGGCAATTATGTCTATTAGTGTTTGTCTCGGAT	36856	0.92
CTBA21	ATCTCGACTAGTCAAAATCTTGGGGGCGTTACGCGGTTGGTGTGGTTGGCATGCTTAGCTGGTCTTCGGTGTCTCGGAT	34741	0.87
CTBA22	ATCTCGACTAGTCAAATTAGATAGGGGGGATTAAGGTTGGTGTGGTTGGCTCTGTTCTCGGCTCGTATGTGTCTCGGAT	33720	0.84
CTBA23	ATCTCGACTAGTCAAGGTAAATAGGGGGCGTCACGGTTGGTGTGGTTGGCATTAGGTCTCTCGTCATTATGTCTCGGAT	31962	0.80
CTBA24	ATCTCGACTAGTCAATTTTAGGGGGCGAAGAAACGGTTGGTGTGGTTGGCGAAATTGTCCATTCGCTATTGTCTCGGAT	30106	0.75
CTBA25	ATCTCGACTAGTCAAGAATGGATATTAGTTTCTCGGTTGGTGTGGTTGGCTTGGGGGCGGCAATAAATATGTCTCGGAT	29468	0.73
CTBA26	ATCTCGACTAGTCAGTTACATAGGGGGCGTTACGCGGTTGGTGTGGTTGGCATCAAGACTGCTCAGTGTTTGTCTCGGAT	28236	0.70
CTBA27	ATCTCGACTAGTCAGGAAAGAGGGGGCGGTGTCTCGGTTGGTGTGGTTGGCTTACGGTGACGTTTAGTTATGTCTCGGAT	26882	0.67
CTBA28	ATCTCGACTAGTCATCTGCAGGGGGCGAAAAAAACGGTTGGTGTGGTTGGCGCGATTAGTCTATCTCTCGTGTCTCGGAT	26878	0.67
CTBA29	ATCTCGACTAGTCATCGTAGGGGGCGGAATCAGCGGTTGGTGTGGTTGGCAAGAATGTATATTGTTAGTTGTCTCGGAT	26672	0.66
CTBA30	ATCTCGACTAGTCATAGAGAAGGGGGCGTCCTGCGGTTGGTGTGGTTGGCATCGGTTATTGTTTAGTATTGTCTCGGAT	25478	0.63
CTBA31	ATCTCGACTAGTCAGAAGGGGGCGGAATTCTCTCGGTTGGTGTGGTTGGCATGAGTGGAAAACTTAATATGTCTCGGAT	25435	0.63
CTBA32	ATCTCGACTAGTCAAGGTCAAGGGGGCAGTCAGTGGTTGGTGTGGTTGGCGACATTTTAATGCGTTATATGTCTCGGAT	25139	0.63
CTBA33	ATCTCGACTAGTCACTGCAGGGGGCGATTTAAACGGTTGGTGTGGTTGGCGCAGTAGTCTCATTATTACTGTCTCGGAT	23538	0.59
CTBA34	ATCTCGACTAGTCAAGAAAATGTCCTTCGGTCTCGGTTGGTGTGGTTGGCTCTGGGGGCGGAACGTATATGTCTCGGAT	23287	0.58
CTBA35	ATCTCGACTAGTCATCTCGTGGGGGCGGTTAAGCGGTTGGTGTGGTTGGCTAAGATTGGTAGGTATAGTTGTCTCGGAT	20806	0.52
CTBA36	ATCTCGACTAGTCACATAGCAAAGGGGGGTCAGGTTGGTGTGGTTGGCTTGCATTTAAGGTCTCGTCTGTCTCGGAT	20620	0.51
CTBA37	ATCTCGACTAGTCAACGAATAGGGGGCGATAAACGGTTGGTGTGGTTGGCATGAGTAGTCATTATATTTTGTCTCGGAT	20121	0.50
CTBA38	ATCTCGACTAGTCAAAGGGGGCGCGGAGACTCGCGGTTGGTGTGGTTGGCGAAACTAGTTTGTTCAATTTGTCTCGGAT	20083	0.50
CTBA39	ATCTCGACTAGTCATCTCGAATAAGGGGGGTCCAGGTTGGTGTGGTTGGCGTTCAATGTTAAAAAAGTCTGTCTCGGAT	19419	0.48
CTBA40	ATCTCGACTAGTCAGTTGTAGCAAGGGGGGATCAAGGTTGGTGTGGTTGGCAGTGTACACGTATATTTAATGTCTCGGAT	19297	0.48
CTBA41	ATCTCGACTAGTCACATGAGGGGGCGAACTTTACGGTTGGTGTGGTTGGCATATGGCTGTCTTTCTTTCTGTCTCGGAT	18486	0.46
CTBA42	ATCTCGACTAGTCATCTAGGAAAAACTTTGTCTCGGTTGGTGTGGTTGGCTCAGGGGGCGGATTAATTTTGTCTCGGAT	17441	0.43
CTBA43	ATCTCGACTAGTCAGTGGGGGCGCGAAATAACGCGGTTGGTGTGGTTGGCATCGTTTGAATCAGTTTTGTGTCTCGGAT	17215	0.43
CTBA44	ATCTCGACTAGTCAAGAAGGGGGCGGCAGAAAGCGGTTGGTGTGGTTGGCTCATAGATTTGTGTTTCATTGTCTCGGAT	15810	0.39
CTBA45	ATCTCGACTAGTCAGGAAAGGGGGCGGTATACTCGGTTGGTGTGGTTGGCTTTGAACTTAAAGTAGCGCTGTCTCGGAT	14408	0.36
CTBA46	ATCTCGACTAGTCATGAAGAAGGGGGCGGTAATCGGTTGGTGTGGTTGGCTCGACTAATGTATGGTTTGTGTCTCGGAT	14312	0.36
CTBA47	ATCTCGACTAGTCAAGGATGATAATTTAGGTCTCGGTTGGTGTGGTTGGCCTCGGGGGCGGATGAAAAGTGTCTCGGAT	14198	0.35
CTBA48	ATCTCGACTAGTCAGGAAAGGGGGCGGTCTCGGCGGTTGGTGTGGTTGGCTTAAGTGTTAAATAATGCATGTCTCGGAT	14115	0.35
CTBA49	ATCTCGACTAGTCAGTTCAAGGGGGCGGAGTTGCGGTTGGTGTGGTTGGCTAATGACATTTGTGCTCGGTGTCTCGGAT	13784	0.34
CTBA50	ATCTCGACTAGTCAGGCAAGTGAGGGGGGATCAAGGTTGGTGTGGTTGGCAACGCGCGTTATTTCTTATTGTCTCGGAT	13649	0.34

Position ruler: 1, 10, 20, 30, 40, 50, 60, 70, 79

Regions: CR1 — RR1 — CR2 — RR2 — CR3

Figure S2. Top 50 sequences from high-throughput sequencing. CR1 (constant region 1), RR1 (random region 1), CR2 (constant region 2), RR2 (random region 2), CR3 (constant region 3). The consecutive G residues are colored in green.

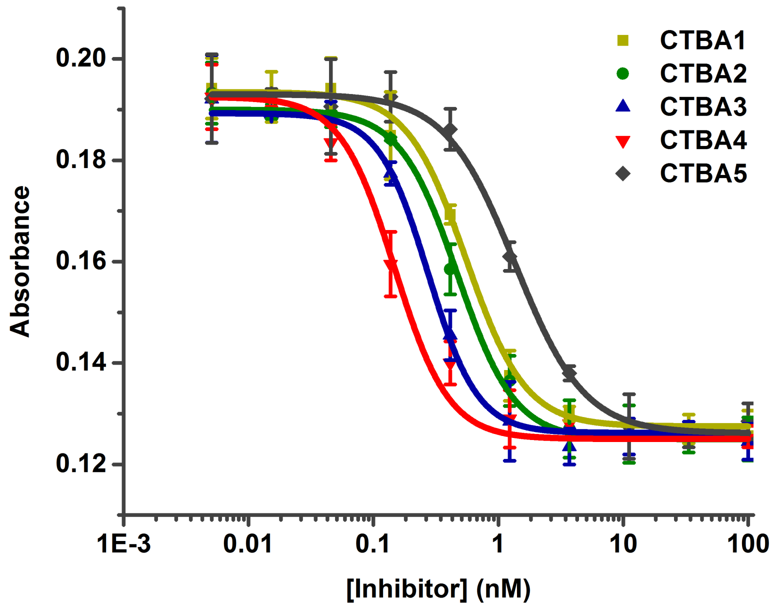

Figure S3. Inhibition of thrombin-induced fibrin polymerization (TIFP) by the top five circular DNA aptamers. The 350-nm absorbance of the thrombin-mediated fibrinogen cleavage reaction mixtures was measured at steady state in the presence of varying concentrations of the listed aptamer candidates as the inhibitory molecules. The data were fitted to a four-parameter logistic model. CTBA4 exhibits the best activity.

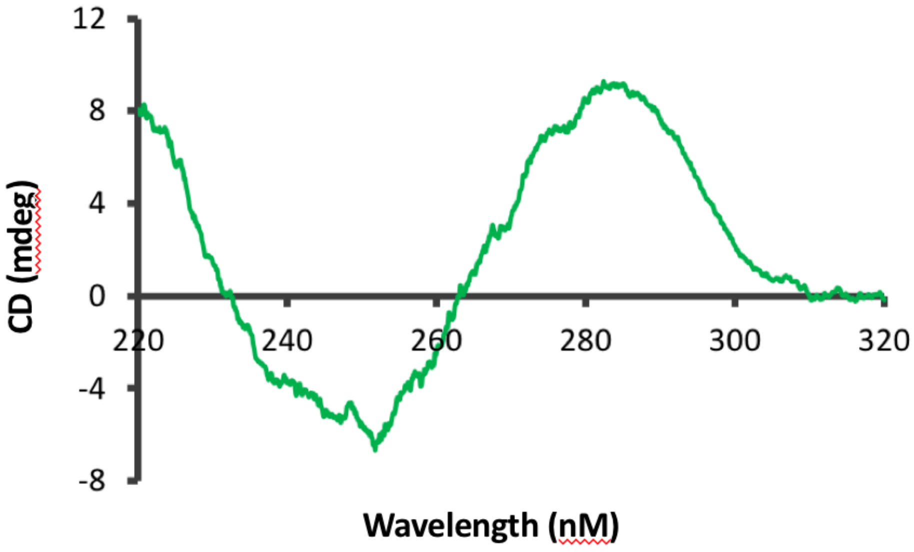

Figure S4. Circular dichroism (CD) analysis of CTBA4T.

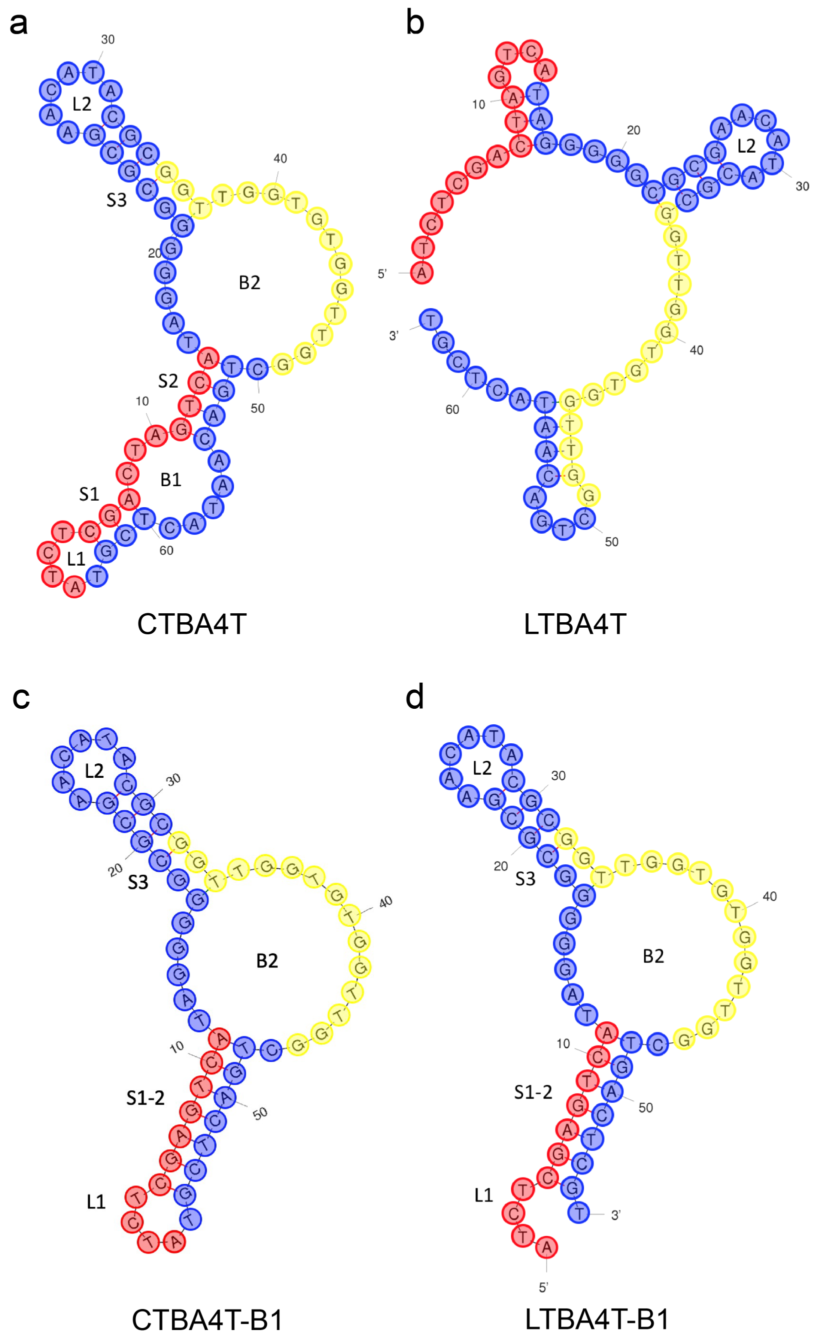

Figure S5. Predicted secondary structures of CTBA4T, LTBA4T, CTBA4T-B1 and LTBA4T-B1 using mfold. Folding conditions: 25 °C, 145 mM NaCl, 1 mM $MgCl_2$. Letters in yellow are nucleotides in the original central constant domain. Letters in blue are nucleotides in the two random-sequence regions. Those in red are nucleotides in the primer domains.

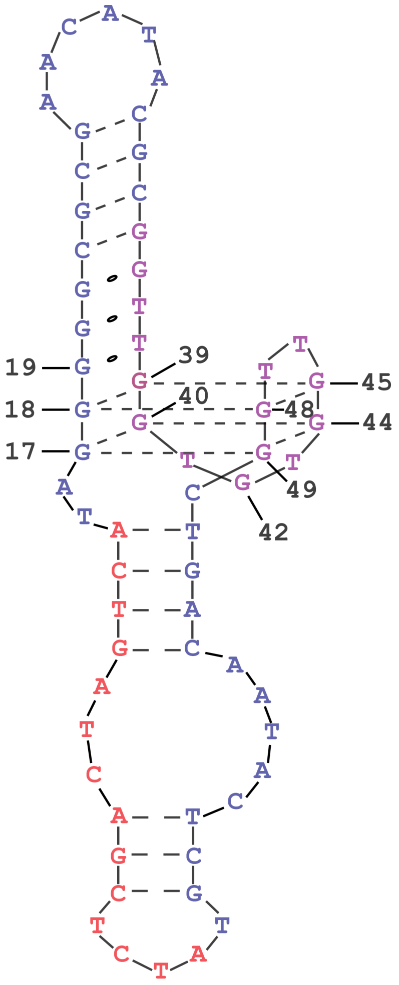

Figure S6. The proposed alternative structure for CTBA4T.

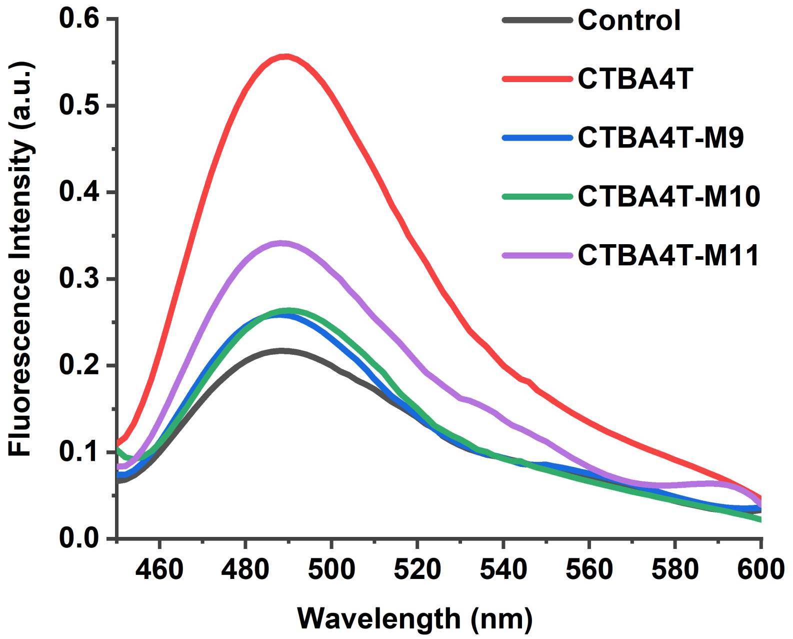

Figure S7. Fluorescence emission spectra of Thioflavin T (ThT) alone and ThT in the presence of CTBA4T and its mutants. The excitation wavelength was set at 425 nm.

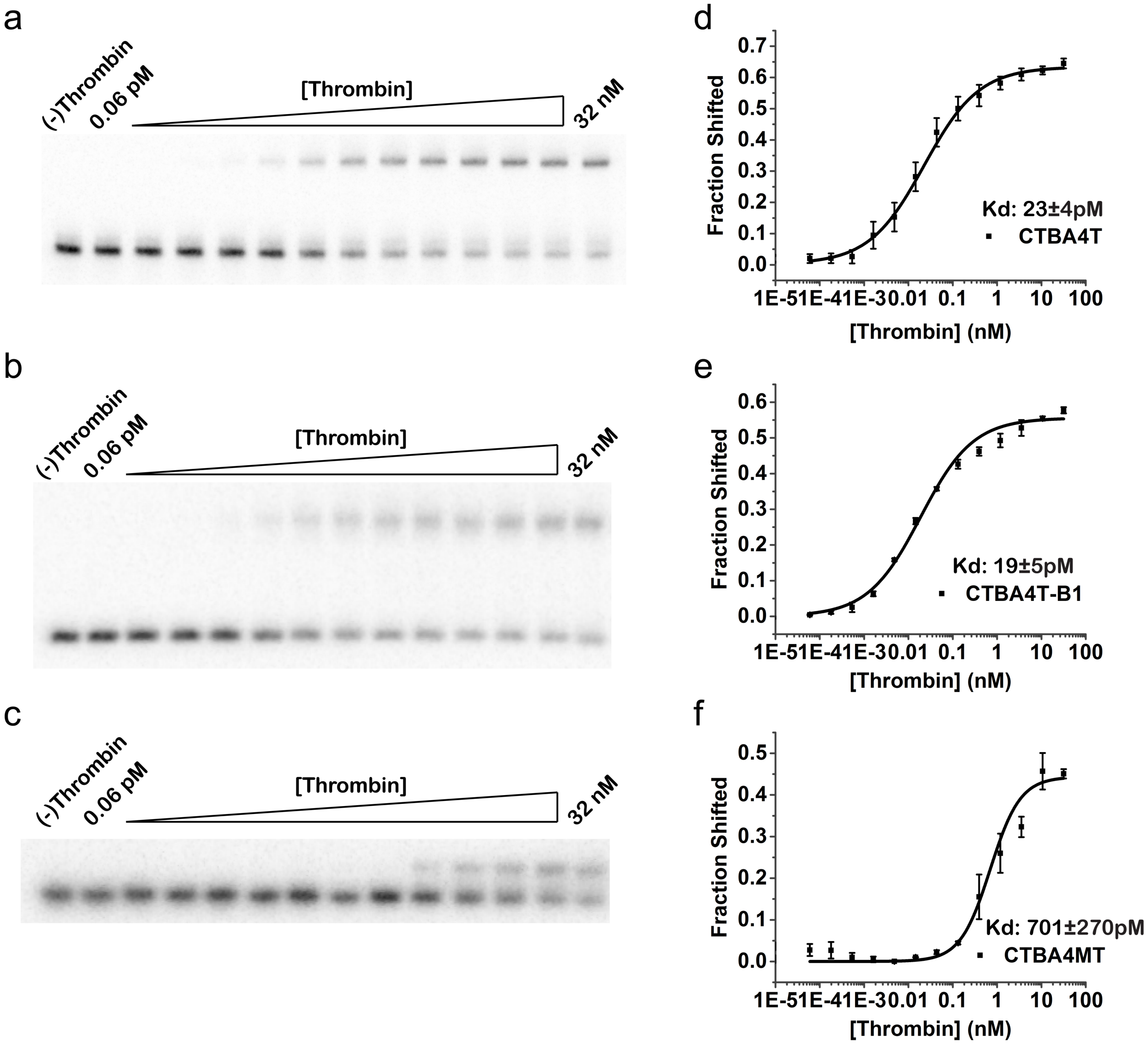

Figure S8. (a-c) EMSA images of radioactively labeled CTBA4T, CTBA4T-B1, and CTBA4MT, respectively, in increasing concentrations of thrombin. (d-f) Fraction of the shifted band (aptamer/thrombin complex) vs. [thrombin] for CTBA4T, CTBA4T-B1, and CTBA4MT, respectively. The data was fitted with the Origin software to a saturation 1:1 binding curve using nonlinear regression, resulting in a K_d value provided in each graph.

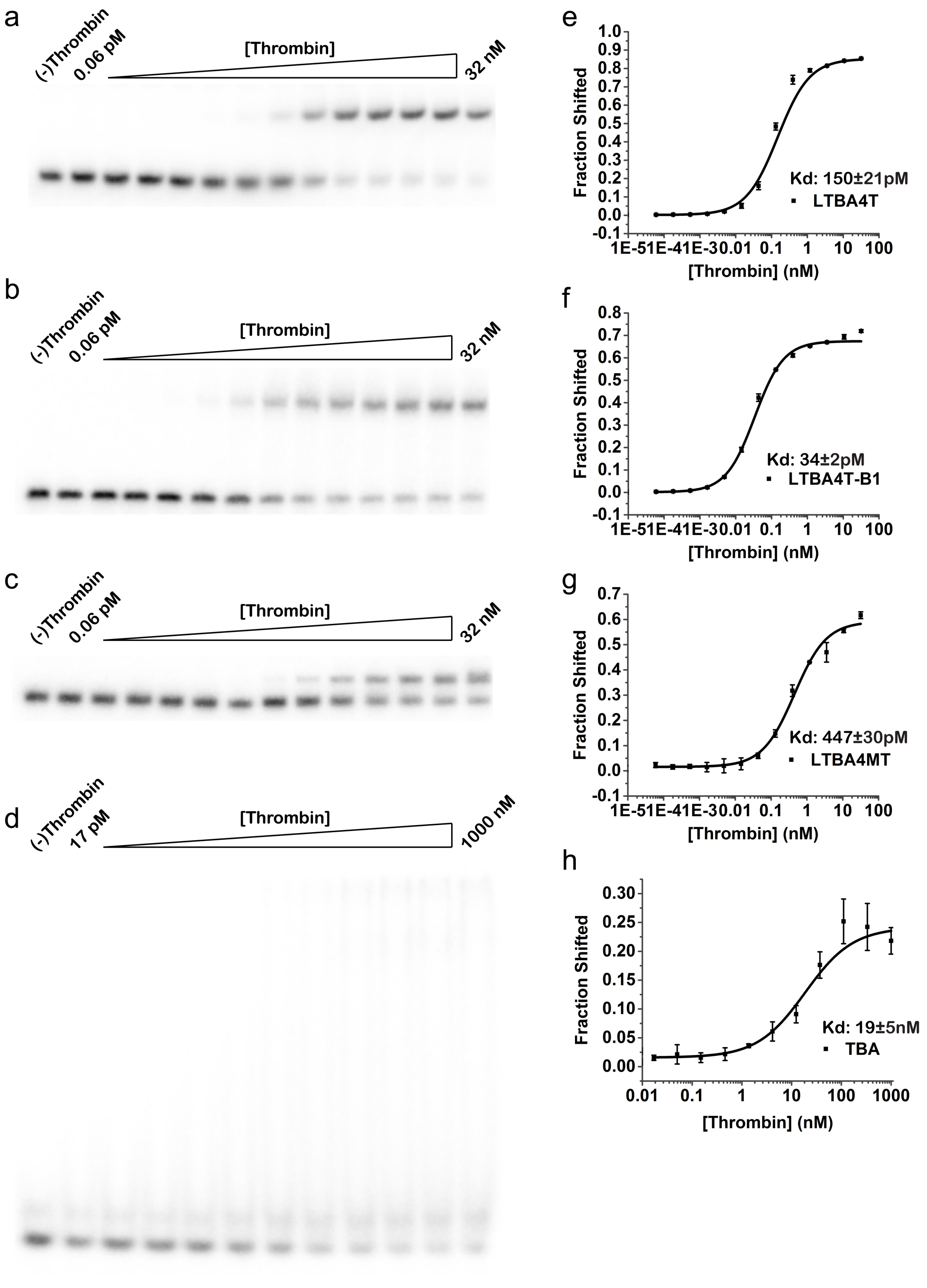

Figure S9. (a-d) EMSA images of radioactively labeled LTBA4T, LTBA4T-B1, LTBA4MT and TBA_{15}, respectively, in increasing concentrations of thrombin. (e-h) Fraction of the shifted band (aptamer/thrombin complex) vs. [thrombin] for LTBA4T, LTBA4T-B1, LTBA4MT and TBA_{15}, respectively. The data was fitted with the Origin software to a saturation 1:1 binding curve using nonlinear regression, resulting in a K_d value provided in each graph.

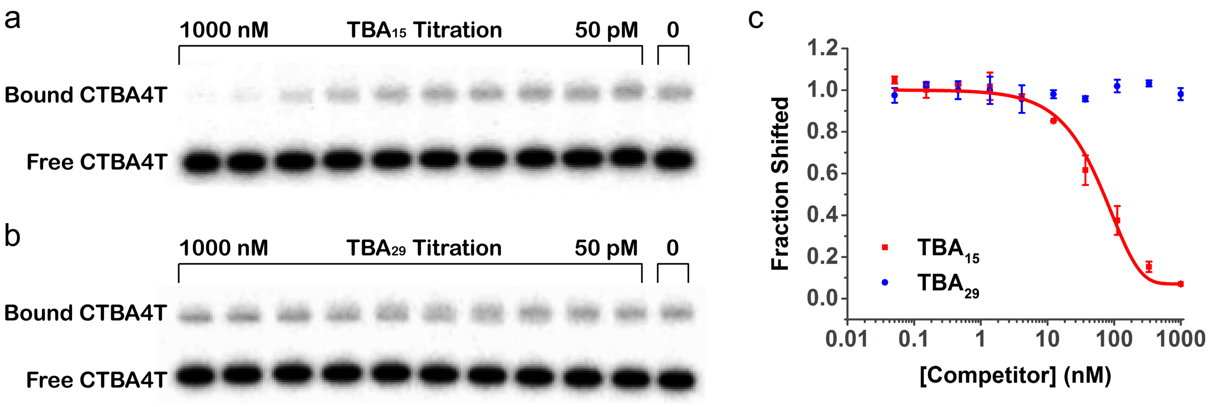

Figure S10. (a) Storage phosphor image for competitive EMSA. CTBA4T + Thrombin vs. TBA_{15}. CTBA4T is radiolabeled. [CTBA4T] = 500 pM, [Thrombin] = 50 pM. (b) Storage phosphor image for competitive EMSA. CTBA4T + Thrombin vs. TBA_{29}. CTBA4T is radiolabeled. [CTBA4T] = 500 pM, [Thrombin] = 50 pM. (c) Plot of fraction shift from CTBA4T + Thrombin vs. Competitor EMSA. Non-linear regression performed for TBA_{15} titration only. No displacement observed for TBA_{29} competitor.

Supplementary Tables

Table S1. Sequences of DNA molecules used in the study.

Name	Sequence (5′-3′)
Lib	ATCTC GACTA GTCAN$_{20}$ GGTTG GTGTG GTTGG N$_{20}$TGTC TCGGA T
LT1	TAGTC GAGAT ATCCG AGACA
LT2	TGTCT CGGAT ATCTC GACTA
CTBA1	ATCTC GACTA GTCAA AAAAA GGGGG CGGGA TCTCG GTTGG TGTGG TTGGC TTTCT GAATA ATCTC GTTAT GTCTC GGAT
CTBA2	ATCTC GACTA GTCAG TGTTA AGGGG GCGGG TCTCG GTTGG TGTGG TTGGC AAATC TCAAT AATGT CTTGT GTCTC GGAT
CTBA3	ATCTC GACTA GTCAA GGGGG CGCGG TCACA TCGCG GTTGG TGTGG TTGGC TGTAA TGGTA CTTCT CGATT GTCTC GGAT
CTBA4	ATCTC GACTA GTCAT AGGGG GCGCG AACAT ACGCG GTTGG TGTGG TTGGC TGACA ATACT CGTTT TTGGT GTCTC GGAT
CTBA5	ATCTC GACTA GTCAA CTGGG GGCGC GAGGA ACCGC GGTTG GTGTG GTTGG CTGTT AAACG TCTTC TGCAT TGTCT CGGAT
CTBA4MT	ATCTC GACTA GTCAT TTTTT TTTTT TTTTT TTTTG GTTGG TGTGG TTGGT TTTTT TTTTT TTTTT TTTTT GTCTC GGAT
CTBA4M1	TTTTT TTCTA GTCAT AGGGG GCGCG AACAT ACGCG GTTGG TGTGG TTGGC TGACA ATACT CGTTT TTGGT GTCTC GGAT
CTBA4M2	ATCTC GATTT TTTTT AGGGG GCGCG AACAT ACGCG GTTGG TGTGG TTGGC TGACA ATACT CGTTT TTGGT GTCTC GGAT
CTBA4M3	ATCTC GACTA GTCAT TTTTT TCGCG AACAT ACGCG GTTGG TGTGG TTGGC TGACA ATACT CGTTT TTGGT GTCTC GGAT
CTBA4M4	ATCTC GACTA GTCAT AGGGG GTTTT TTTAT ACGCG GTTGG TGTGG TTGGC TGACA ATACT CGTTT TTGGT GTCTC GGAT
CTBA4M5	ATCTC GACTA GTCAT AGGGG GCGCG AACTT TTTTT GTTGG TGTGG TTGGC TGACA ATACT CGTTT TTGGT GTCTC GGAT
CTBA4M6	ATCTC GACTA GTCAT AGGGG GCGCG AACAT ACGCG TTTTT TTTGG TTGGC TGACA ATACT CGTTT TTGGT GTCTC GGAT
CTBA4M7	ATCTC GACTA GTCAT AGGGG GCGCG AACAT ACGCG GTTGG TGTTT TTTTC TGACA ATACT CGTTT TTGGT GTCTC GGAT
CTBA4M8	ATCTC GACTA GTCAT AGGGG GCGCG AACAT ACGCG GTTGG TGTGG TTGGT TTTTT TTACT CGTTT TTGGT GTCTC GGAT
CTBA4M9	ATCTC GACTA GTCAT AGGGG GCGCG AACAT ACGCG GTTGG TGTGG TTGGC TGACA ATTTT TTTTT TTGGT GTCTC GGAT
CTBA4M10	ATCTC GACTA GTCAT AGGGG GCGCG AACAT ACGCG GTTGG TGTGG TTGGC TGACA ATACT CGTTT TTTTT GTCTC GGAT
CTBA4M11	ATCTC GACTA GTCAT AGGGG GCGCG AACAT ACGCG GTTGG TGTGG TTGGC TGACA ATACT CGTTT TTGGT TTTTT TTTT
CTBA4T	ATCTC GACTA GTCAT AGGGG GCGCG AACAT ACGCG GTTGG TGTGG TTGGC TGACA ATACT CGT
CTBA4T-M1	ATCTC GACTA GTCAT AGGGG GCGCG TTTTA TCGCG GTTGG TGTGG TTGGC TGACA ATACT CGT
CTBA4T-M2	ATCTC GACTA GTCAT AGGGG GCGCG AACAT ACGCG TTTGG TGTGG TTGGC TGACA ATACT CGT
CTBA4T-M3	ATCTC GACTA GTCAT AGGGG GCGCG AACAT ACGCT GTTGG TGTGG TTGGC TGACA ATACT CGT
CTBA4T-M4	ATCTC GACTA GTCAT AGGGG GCCGC AACAT AGCGG GTTGG TGTGG TTGGC TGACA ATACT CGT
CTBA4T-M5	ATCTC GACTA GTCAT AGGGG GCGCG AACAT ACTCG GTTGG TGTGG TTGGC TGACA ATACT CGT

CTBA4T-M6	ATCTC GACTA GTCAT AGGGG GTGCG AACAT ACGCG GTTGG TGTGG TTGGC TGACA ATACT CGT
CTBA4T-M7	ATCTC GACTA GTCAT AGGGG TCGCG AACAT ACGCG GTTGG TGTGG TTGGC TGACA ATACT CGT
CTBA4T-M8	ATCTC GACTA GTCAT AGGGT GCGCG AACAT ACGCG GTTGG TGTGG TTGGC TGACA ATACT CGT
CTBA4T-M9	ATCTC GACTA GTCAT AGTGG GCGCG AACAT ACGCG GTTGG TGTGG TTGGC TGACA ATACT CGT
CTBA4T-M10	ATCTC GACTA GTCAT AGGTG GCGCG AACAT ACGCG GTTGG TGTGG TTGGC TGACA ATACT CGT
CTBA4T-M11	ATCTC GACTA GTCAT ATGGG GCGCG AACAT ACGCG GTTGG TGTGG TTGGC TGACA ATACT CGT
CTBA4T-M12	ATCTC GACTA GTCAT TGGGG GCGCG AACAT ACGCG GTTGG TGTGG TTGGC TGACA ATACT CGT
CTBA4T-M13	ATCTC GACTA GTCAC AGGGG GCGCG AACAT ACGCG GTTGG TGTGG TTGGC TGACA ATACT CGT
CTBA4T-M14	ATCTC GACTA GTCAT AGGGG GCGCG AACAT ACGCG GTTGG TGTGG TTGGT TGACA ATACT CGT
CTBA4T-B1	ATCTC GAGTC ATAGG GGGCG CGAAC ATACG CGGTT GGTGT GGTTG GCTGA CTCGT
FP	GCCTC AACTT ATCCG AGACA
RP	GGCAC TATCT CGACT AGTCA
TBA_{15}	GGTTG GTGTG GTTGG
TBA_{29}	AGTCC GTGGT AGGGC AGGTT GGGGT GACT

Table S2. High-throughput sequencing results from the round 7 pool.

ID	Multiplicity	% of Total
CTBA1	338573	8.43
CTBA2	278871	6.94
CTBA3	278053	6.92
CTBA4	226305	5.64
CTBA5	111078	2.77

Table S3. Inhibition of thrombin-induced fibrin polymerization (TIFP) of the top five circular aptamers as measured by IC_{50}.

Circular aptamer candidate	IC_{50} (nM)
CTBA1	0.59 ± 0.07
CTBA2	0.48 ± 0.06
CTBA3	0.30 ± 0.01
CTBA4	0.15 ± 0.01
CTBA5	1.55 ± 0.25

Table S4. Melting temperatures.

Oligonucleotide	T_m (°C)
TBA_{15}	50 ± 3
LTBA4T	53 ± 2
CTBA4T	65 ± 2
LTBA4T-B1	62 ± 2
CTBA4T-B1	72 ± 1
